# Potential Benefits of Sodium-Glucose Transporter-2 Inhibitors in the Symptomatic and Functional Status of Patients With Heart Failure: A Systematic Review and Meta-Analysis

**DOI:** 10.7759/cureus.29579

**Published:** 2022-09-25

**Authors:** Sushen Bhalla, Yousif AlQabandi, Savitri Aninditha Nandula, Chinmayi Sree Boddepalli, Sai Dheeraj Gutlapalli, Vamsi Krishna Lavu, Rana Abdelwahab Mohamed Abdelwahab, Ruimin Huang, Shanthi Potla, Pousette Hamid

**Affiliations:** 1 Internal Medicine, California Institute of Behavioral Neurosciences & Psychology, Fairfield, USA; 2 Ophthalmology, California Institute of Behavioral Neurosciences & Psychology, Fairfield, USA; 3 Dermatology, California Institute of Behavioral Neurosciences & Psychology, Fairfield, USA; 4 Psychiatry, California Institute of Behavioral Neurosciences & Psychology, Fairfield, USA; 5 Neurology, California Institute of Behavioral Neurosciences & Psychology, Fairfield, USA

**Keywords:** cardiovascular mortality, hospitalization for heart failure, all-cause mortality, functional improvement, quality of life, heart failure, sodium-glucose transporter-2 inhibitors

## Abstract

This review evaluates the potential benefits of sodium-glucose transporter-2 (SGLT-2) inhibitors on symptom burden/health-related quality of life (HRQoL), functional improvement, hospitalization for heart failure (HHF), cardiovascular mortality (CVM), and all-cause mortality (ACM) in patients with heart failure (HF) with reduced or preserved ejection fraction (EF). We analyzed 12 randomized clinical trials (RCTs) accessed through 11 records and three secondary analyses from PubMed and Scopus following Preferred Reporting Items for Systematic Reviews and Meta-analyses (PRISMA) 2020 guidelines. These studies evaluated 23,389 patients treated with either an SGLT-2 inhibitor or placebo in addition to the standard of care. Four studies recruited diabetic patients, some of whom had HF at the baseline and were evaluated as a subgroup. One study had diabetes and HF present in all patients at the baseline. Ten studies recruited patients with HF at their baseline irrespective of diabetic status. Eight studies evaluated the SGLT-2 inhibitors for a composite of hospitalization for heart failure or cardiovascular mortality (HHF/CVM) and ACM. Five of these studies showed a decreased risk for HHF/CVM, and two showed a reduced risk for ACM. One trial showed benefits in patients with heart failure with reduced ejection fraction (HFrEF) only and not in heart failure with preserved ejection fraction (HFpEF). Other studies revealed benefits but did not reach statistical significance. Ten studies assessed the SGLT-2 inhibitors for improvement in symptoms and HRQoL; four demonstrated a significant improvement, three showed a slight improvement, and three did not find any benefit. Five trials evaluated participants’ functional progress by assessing for a six-minute walk test (6MWT). Two studies showed a significant increase in the distance walked by the patient, while three others did not. The SGLT-2 inhibitors reduce the risk of HHF/CVM irrespective of ejection fraction and result in a symptomatic improvement.

## Introduction and background

Most cardiac pathologies, and many that do not directly involve the heart, e.g., diabetes mellitus or obesity, may result in heart failure (HF) as the end-stage phenotypic expression of the primary disease [1]. According to one study, the HF prevalence in the United States of America and Canada lies between 1.5%-1.9% and 1%-2% in the European population [1]. Other studies estimated that 64.3 million people suffered from HF globally in 2017, with the highest prevalence in Central Europe, the Middle East, and North Africa and the lowest rates in Eastern Europe and Southeast Asia [2]. Heart failure patients carry a post-hospitalization five-year case fatality rate of about 42%, with higher incidence, prevalence, and mortality rates in females [3]. About one-third to half of all HF patients have heart failure with preserved ejection fraction (HFpEF) [4,5].

The institution of new therapeutic guidelines based on the antagonism of neurohormonal pathophysiology involving beta-blockers, angiotensin-converting enzyme inhibitors (ACEI) [6], angiotensin receptor blockers (ARB), angiotensin receptor and neprilysin inhibitor (ARNI) [7], and mineralocorticoid receptor antagonists (MRA) has reduced the mortality and morbidity over the last few decades. However, this benefit was primarily restricted to heart failure with reduced ejection fraction (HFrEF) and not HFpEF [8]. This difference may have been secondary to the heterogeneity of the HFpEF clinical syndrome as reflected by the pathophysiological role of comorbidities, e.g., hyperinsulinemia in non-insulin-dependent diabetes mellitus as the cause of cardiac remodeling [9], elevated afterload leading to left ventricular (LV) hypertrophy in hypertension [10], the presence of epicardial adipose tissue resulting in restricted relaxation of the left chambers [11], and age-related fibrotic changes in older patients [12].

The advent of sodium-glucose transporter-2 (SGLT-2) inhibitors for the treatment of diabetes mellitus showed a reduction in hospitalization for heart failure (HHF) in HFrEF and HFpEF patients [13-16]. The data, however, led to unclear results for other vital endpoints having a bearing on the patient’s life, e.g., cardiovascular mortality (CVM), all-cause mortality (ACM), and health-related quality of life (HRQoL) [13-15]. One reason for the lack of statistical significance is the low power of each study, as these factors are studied as secondary endpoints. From the perspective of both the patient and the clinician, it is essential to understand the impact of SGLT-2 inhibitors on these endpoints.

We performed a comprehensive review of randomized clinical trials (RCTs) evaluating SGLT-2 inhibitors in HF patients with different values of ejection fraction (EF) to create a more lucid concept of these interventions on patients’ survival and quality of life besides the worsening of heart failure.

## Review

Methodology

We followed the Preferred Reporting Items for Systematic Reviews and Meta-Analyses (PRISMA) 2020 guidelines to conduct this systematic review and meta-analysis [17].

Question

We needed to answer the question if sodium-glucose transporter-2 (SGLT-2) inhibitors provide any beneficial role in symptomatic burden, functional status, the composite of hospitalization for heart failure or cardiovascular mortality (HHF/CVM), and all-cause mortality (ACM) in patients with heart failure with reduced ejection fraction (HFrEF) or heart failure with preserved ejection fraction (HFpEF). The safety concerns included urinary tract infection (UTI), hypovolemia, acute kidney injury (AKI), amputation, and fracture. 

Inclusion/exclusion criteria

We selected peer-reviewed randomized clinical trials (RCTs) and sub-analyses of the trials with patients having heart failure (HF) at the baseline, comparing SGLT-2 inhibitors and placebo for the specified outcomes. We excluded observational studies, reviews, editorials, meta-analyses, and studies with patients in acute HF.

Data extraction

A systematic literature search was conducted in online databases of PubMed and Scopus on May 24, 2022. We used the following search terms in combination: “Heart failure,” “Sodium glucose cotransporter 2 inhibitors,” “sglt2 inhibitors,” “Canagliflozin,” “Dapagliflozin,” “Empagliflozin,” “Ertugliflozin,” “Ipragliflozin,” “Licogliflozin,” “Luseogliflozin,” “Sotagliflozin,” and “Tofogliflozin.” On PubMed, Medical Subject Headings (MeSH) search strategy was used: (“Heart Failure” {Majr}) OR “Heart Failure” (Mesh:NoExp) AND (“Sodium-Glucose Transporter 2 Inhibitors” {Majr}) OR “Sodium-Glucose Transporter 2 Inhibitors” (Mesh:NoExp) OR (“Canagliflozin” {Majr}) OR “Canagliflozin” (Mesh:NoExp) OR “dapagliflozin” (Supplementary Concept) OR “empagliflozin” (Supplementary Concept) OR “ertugliflozin” (Supplementary Concept) OR “ipragliflozin” (Supplementary Concept) OR (“licogliflozin” {Majr}) OR “licogliflozin” (Supplementary Concept:NoExp) OR “1,5-anhydro-1-(5-(4-ethoxybenzyl)-2-methoxy-4-methylphenyl)-1-thioglucitol” (Supplementary Concept) OR “(2S,3R,4R,5S,6R)-2-(4-chloro-3-(4-ethoxybenzyl)phenyl)-6-(methylthio)tetrahydro-2H-pyran-3,4,5-triol” (Supplementary Concept) OR “6-((4-ethylphenyl)methyl)-3',4',5',6'-tetrahydro-6'-(hydroxymethyl)spiro(isobenzofuran-1(3H),2'-(2H)pyran)-3',4',5'-triol” (Supplementary Concept). The filters used were for the English-language RCTs conducted on humans over the last ten years with the full text of interest available. We checked the references of other reviews and meta-analyses for studies that might not have been shown in the search.

**Figure 1:**
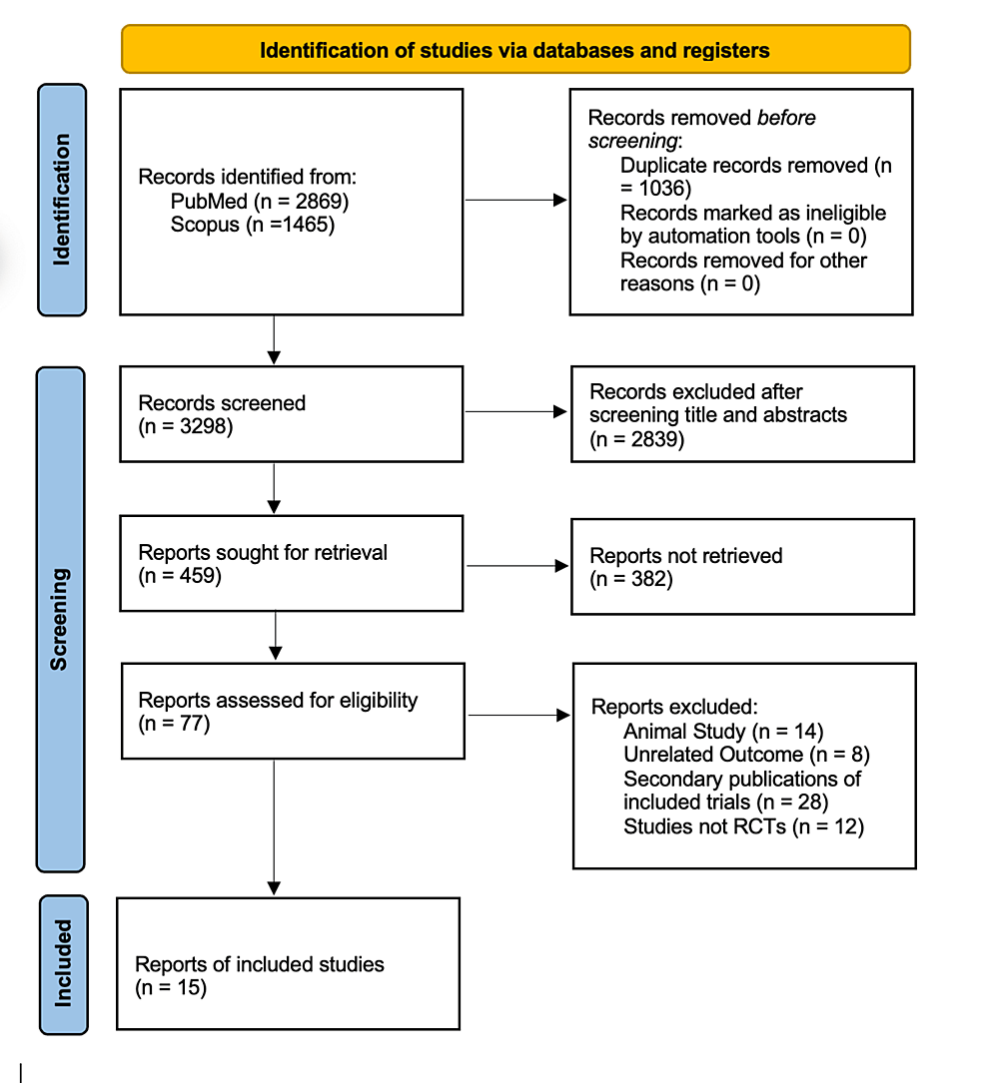
Preferred Reporting Items for Systematic Reviews and Meta-Analyses (PRISMA) flow diagram RCTs: randomized clinical trials

After applying the filters, we accessed 2,869 and 1,465 articles from PubMed and Scopus, respectively. We removed 1,036 duplicates and 2,839 articles by screening the titles and abstracts (Figure 1). After reviewing the full articles, we excluded 382 articles that could not be retrieved and 62 that were not relevant to our study. We included 15 articles that matched our inclusion/exclusion criteria for data extraction.

The assessment for quality and potential for bias was carried out by two reviewers (SB and YQ) independently with Cochrane risk bias assessment tools for clinical trials [18] based on six domains, as shown in Table 1, and discrepancies were resolved with mutual discussion.

**Table 1:** Quality assessment of selected studies

Author, year, study	Random sequence generation	Allocation concealment	Blinding of patients and personnel	Blinding of outcome assessment	Incomplete outcome data	Selective reporting
Cosentino et al., 2020, VERTIS-CV [19]	Low	Low	Low	Unclear	Low	Low
Bhatt et al., 2021, SOLOIST-WHF [20]	Low	Low	Low	Low	Unclear	Low
Nassif et al., 2021, PRESERVED-HF [21]	Unclear	Unclear	Low	Low	Low	Low
Nassif et al., 2019, DEFINE-HF [22]	Unclear	Unclear	Unclear	Low	Low	Low
Abraham et al., 2021, EMPERIAL [23]	Low	Low	Low	Low	Low	Low
Santos-Gallego et al., 2021, EMPA-TROPISM [24]	Low	Low	Low	Low	Low	Low
Jensen et al., 2020, EMPIRE-HF [25]	Low	Low	Low	Low	Low	Low
Lee et al., 2021, SUGAR-DM-HF [26]	Low	Low	Low	Low	Low	Low
Packer et al., 2020, EMPEROR Reduced [27]	Low	Low	Low	Unclear	Low	Low
Anker et al., 2021, EMPEROR Preserved [28]	Low	Low	Low	Low	Low	Low
McMurray et al., 2019, DAPA-HF [29]	Low	Low	Low	Low	Low	Low

Characteristics of the included studies

We evaluated the results of 23,389 patients who participated in 12 randomized clinical trials and three post hoc analyses with a follow-up period range of 3-50.4 months. The demographic characteristics of the patients included in these studies are shown in Table 2.

**Table 2:** Demographic characteristics of the patients included in this review SGLT-2i: sodium-glucose transporter-2 inhibitor; n: number of patients; DM: diabetes mellitus; FU: follow-up

Author, year, study	SGLT-2i	n	Age, mean	DM, %	FU months
Cosentino et al., 2020, VERTIS-CV [19]	Ertugliflozin	1,958	64.4	100	42
Bhatt et al., 2021, SOLOIST [20]	Sotagliflozin	1,222	70	100	9
Nassif et al., 2021, PRESERVED-HF [21]	Dapagliflozin	324	70	56	3
Nassif et al., 2019, DEFINE-HF [22]	Dapagliflozin	263	61.3	63	3
Abraham et al., 2021, EMPERIAL-Reduced [23]	Empagliflozin	312	69.5	60	3
Abraham et al., 2021, EMPERIAL-Preserved [23]	Empagliflozin	315	74	51	3
Santos-Gallego et al., 2021, EMPA-TROPISM [24]	Empagliflozin	84	62	0	6
Jensen et al., 2020, EMPIRE-HF [25]	Empagliflozin	190	64	18	3
Lee et al., 2021, SUGAR-DM-HF [26]	Empagliflozin	105	68.7	100	9
Packer et al., 2020, EMPEROR-Reduced [27]	Empagliflozin	3,730	67.2	49.8	16
Anker et al., 2021, EMPEROR-Preserved [28]	Empagliflozin	5,988	71.9	49	26.2
McMurray et al., 2019, DAPA-HF [29]	Dapagliflozin	4,744	66.2	42	18.2
Rådholm et al., 2018, CANVAS [30]	Canagliflozin	1,461	63.8	100	47
Kato et al., 2019, DECLARE-TIMI 58 [31]	Dapagliflozin	1,987	63.9	100	50.4
Fitchett et al., 2016, EMPA-REG Outcome [32]	Empagliflozin	706	64.5	100	37.2

The cardiovascular and renal parameters of the patients included in these studies are shown in Table 3.

**Table 3:** Cardiovascular and renal parameters of the patients included in this review NYHA: New York Heart Association; LVEF: left ventricular ejection fraction; NT-pro BNP: N-terminal pro b-type natriuretic peptide; pg/ml: picogram/milliliter; eGFR: estimated glomerular filtration rate; ml/min: milliliter/minute; n: number of patients; EF: ejection fraction; NA: not available

Author, year, study	NYHA class, %				LVEF, mean, %	NT-pro BNP, pg/ml	eGFR, ml/min/1.73 m^2^, mean
	I	II	III	IV			
Cosentino et al., 2020, VERTIS-CV [19]	24	66	7	0	n = 1007: EF >45, n = 478: EF <45	NA	NA
Bhatt et al., 2021, SOLOIST [20]	2	46	47	4.4	n = 256: EF ≥50, n = 966: EF <50	1,800	49.7
Nassif et al., 2021, PRESERVED-HF [21]	0	58	42		60	675	55
Nassif et al., 2019, DEFINE-HF [22]	0	66	34	0	27	1,136	69
Abraham et al., 2021, EMPERIAL-Reduced [23]	0	65	35	0	30	1,489	55
Abraham et al., 2021, EMPERIAL-Preserved [23]	0	77	23	0	53	898	57
Santos-Gallego et al., 2021, EMPA-TROPISM [24]	NA				36	NA	81.5
Jensen et al., 2020, EMPIRE-HF [25]	6	79	15	0	30	594	74
Lee et al., 2021, SUGAR-DM-HF [26]	0	77	23	0	32.5	466	67.3
Packer et al., 2020, EMPEROR-Reduced [27]	0	75	24	1	28	1,906	62
Anker et al., 2021, EMPEROR-Preserved [28]	0.1	81	18	0.4	54	970	60.6
McMurray et al., 2019, DAPA-HF [29]	0	67	32	1	31	1,437	65.7
Rådholm et al., 2018, CANVAS [30]	NA				NA	NA	73.0
Kato et al., 2019, DECLARE-TIMI 58 [31]	35	56	8.5	0.5	n = 808: EF ≥45, n = 671: EF <45	NA	85
Fitchett et al., 2016, EMPA-REG Outcome [32]	NA				NA	NA	69

Four trials (VERTIS-CV, CANVAS, DECLARE-TIMI 58, and EMPA-REG Outcome) recruited diabetic patients with 14.4%, 11.6%, 10.1%, and 23.7% of the cohort having heart failure (HF) at the baseline [19,30-32]. The CANVAS [30] and EMPA-REG Outcome [32] trials had patients with a high risk of cardiovascular disease (CVD), but those with HF were not investigated for ejection fraction (EF) at the baseline. The VERTIS-CV, SOLOIST, and DECLARE-TIMI 58 trials had patients with heart failure with reduced ejection fraction (HFrEF) and heart failure with preserved ejection fraction (HFpEF) [19,20,31]. The SOLOIST trial had participants admitted for decompensated heart failure, and the intervention is an inhibitor of both sodium-glucose transporter-2 and sodium-glucose transporter-1 channels [20]. The trials PRESERVED-HF, EMPERIAL-Preserved, and EMPEROR-Preserved recruited patients with only HFpEF with a mean EF of >50% [21,23,28]. Patients with HFrEF in the rest of the trials had a mean EF of <40%.

Results

The results of the CANVAS trial [30] showed a decrease in the composite of hospitalization for heart failure or cardiovascular mortality (HHF/CVM) but not a significant reduction in all-cause mortality (ACM) for these patients. The benefit was more remarkable for patients with heart failure (HF) than those with no HF at baseline. The subgroup of patients with HF in the DECLARE-TIMI 58 [31] trial was stratified into two groups based on the ejection fraction (EF) cutoff of 45%. All patients benefited for HHF/CVM risk, and a significantly decreased risk of ACM was observed in patients with heart failure with reduced ejection fraction (HFrEF). The EMPA-REG Outcome trial [32] showed a significant fall in HHF/CVM and ACM in the overall cohort. Still, the same benefit did not achieve statistical significance in the subgroup of patients with heart failure. Using ertugliflozin in the VERTIS-CV [19] trial demonstrated a delay to the first hospitalization for heart failure (HHF), risk of total HHF, and total HHF/CVM in the overall cohort.

The SOLOIST trial [20] showed a significant difference in the two groups favoring sotagliflozin over placebo for CVM and HHF risk, which held for both reduced and preserved EFs, but there was no benefit for ACM. This study recruited patients admitted for decompensated HF who were started on sotagliflozin either in the hospital or soon after.

The DAPA-HF study [29] demonstrated a risk reduction in HHF/CVM and ACM. The participants in the EMPEROR-Reduced trial [27] had an EF between 30% and 40%, and the results reflected a decreased risk of a composite of HHF/CVM. There was a non-significant decrease in ACM. Empagliflozin also improved HHF/CVM risk in the EMPEROR-Preserved [28] trial across different EFs from 40% to >60% but had no effect on ACM. These results are shown in Table 4.

**Table 4:** Results of risks of HHF/CVM and CVM in respective trials CI: confidence interval; CVM: cardiovascular mortality; HHF: hospitalization for heart failure; P: P value; ACM: all-cause mortality; HFrEF: heart failure with reduced ejection fraction; HFpEF: heart failure with preserved ejection fraction; NA: not available

Author, year, study	Hazard ratio (95% CI) for CVM or HHF	P	Hazard ratio (95% CI) for ACM	P
Cosentino et al., 2020, VERTIS-CV [19]	HFrEF: 0.76 (0.51-1.14)	0.74	HFrEF: 0.96 (0.61-1.53)	0.98
Cosentino et al., 2020, VERTIS-CV [19]	HFpEF: 0.92 (0.61-1.39)	0.74	HFpEF: 1.01 (0.66-1.56)	0.98
Bhatt et al., 2021, SOLOIST-WHF [20]	All patients: 0.67 (0.52-0.85)	<0.001	0.82 (0.59-1.14)	NA
Bhatt et al., 2021, SOLOIST-WHF [20]	HFrEF: 0.72 (0.56-0.94)	<0.001	0.82 (0.59-1.14)	NA
Bhatt et al., 2021, SOLOIST-WHF [20]	HFpEF: 0.48 (0.27-0.86)	<0.001	0.82 (0.59-1.14)	NA
Packer et al., 2020, EMPEROR-Reduced [27]	0.75 (0.65-0.86)	<0.001	0.92 (0.77-1.10)	NA
Anker et al., 2021, EMPEROR-Preserved [28]	0.79 (0.69-0.90)	<0.001	1.00 (0.87-1.15)	NA
McMurray et al., 2019, DAPA-HF [29]	0.75 (0.65-0.85)	<0.001	0.83 (0.71-0.97)	NA
Rådholm et al., 2018, CANVAS [30]	0.61 (0.46-0.80)	0.02	0.70 (0.51, 0.96)	0.16
Kato et al., 2019, DECLARE-TIMI 58 [31]	HFrEF: 0.62 (0.45-0.86)	0.046	HFrEF: 0.59 (0.40-0.88)	0.016
Kato et al., 2019, DECLARE-TIMI 58 [31]	HFpEF: 0.88 (0.66-1.17)	0.046	HFpEF: 1.02 (0.75-1.38)	0.016
Fitchett et al., 2016, EMPA-REG Outcome [32]	0.72 (0.50-1.04)	NA	0.79 (0.52-1.20)	NA

The SOLOIST trial [20] showed a 4.1-point improvement in the intervention group’s Kansas City Cardiac Questionnaire-12 (KCCQ-12) score. The DAPA-HF [29] study demonstrated symptomatic improvement at the end of eight months with an increase in the Kansas City Cardiac Questionnaire-total summary score (KCCQ-TSS). Empagliflozin provided a small improvement in the symptom burden of patients with reduced and preserved ejection fractions in EMPEROR-Reduced and EMPEROR-Preserved trials [27,28]. The PRESERVED-HF trial recruited HF patients with a mean EF of 60% and reported an improvement in the Kansas City Cardiac Questionnaire-clinical summary score (KCCQ-CSS), Kansas City Cardiac Questionnaire-overall summary score (KCCQ-OSS), and KCCQ-TSS at 12 weeks [21]. Dapagliflozin also showed an improvement in symptoms in HFrEF patients (EF <40%) in the DEFINE-HF trial [22] with an increase in the KCCQ scores in all domains. The EMPERIAL-Preserved [23] trial did not find similar symptomatic improvement in patients with either reduced or preserved ejection fraction.

The EMPA-TROPISM trial [24] investigated the effect of empagliflozin in nondiabetic patients with HFrEF for reducing left ventricular (LV) end-diastolic and end-systolic volumes and LV mass and improving quality of life with better symptomatic control. In contrast, the same molecule showed no symptomatic improvement in either the EMPIRE-HF or SUGAR-DM-HF trials [25,26]. These results are shown in Table 5.

**Table 5:** Results of symptomatic improvement of respective trials KCCQ-12: Kansas City Cardiac Questionnaire-12; SGLT-2i: sodium-glucose transporter-2 inhibitor; KCCQ-CSS: Kansas City Cardiac Questionnaire-clinical summary score; KCCQ-TSS: Kansas City Cardiac Questionnaire-total symptom score; KCCQ-OSS: Kansas City Cardiac Questionnaire-overall summary score;* *P: P value; EF: ejection fraction

Author, year, study	KCCQ-12 score difference between SGLT-2i and placebo with 95% confidence interval
Bhatt et al., 2021, SOLOIST-WHF [20]	KCCQ-12: 4.1 (1.3-7.0)
Nassif et al., 2021, DAPA-PRESERVED [21]	KCCQ-CSS: 5.8 (2.3-9.2), P = 0.001
Nassif et al., 2021, DAPA-PRESERVED [21]	KCCQ-TSS: 5.8 (2.0-9.6), P = 0.003
Nassif et al., 2021, DAPA-PRESERVED [21]	KCCQ-OSS: 4.5 (1.1-7.8), P = 0.009
Nassif et al., 2019, DEFINE-HF [22]	KCCQ-OSS: 3.7, P = 0.037
Nassif et al., 2019, DEFINE-HF [22]	KCCQ-CSS: 4.6,P = 0.007
Nassif et al., 2019, DEFINE-HF [22]	KCCQ-TSS: 4.8, P = 0.012
Abraham et al., 2021, EMPERIAL [23]	KCCQ-TSS: 3.13 (0.00-7.29) for EF <40%
Abraham et al., 2021, EMPERIAL [23]	KCCQ-TSS: 2.08 (-2.08-6.25) for EF >40%
Santos-Gallego et al., 2021, EMPA-TROPISM [24]	KCCQ-12: 21 ± 18 (empagliflozin) versus 2 ± 15 (placebo), P < 0.001
Jensen et al., 2020, EMPIRE-HF [25]	KCCQ-CSS: 3.1 (-0.2-6.4),P = 0.07
Jensen et al., 2020, EMPIRE-HF [25]	KCCQ-TSS: 2.3 (-1.0-5.6), P = 0.20
Lee et al., 2021, SUGAR-DM-HF [26]	KCCQ-TSS: -4.0 (-10.2-2.1), P = 0.19
Packer et al., 2020, EMPEROR-Reduced [27]	KCCQ-12: 1.7 (0.5-3.0)
Anker et al., 2021, EMPEROR-Preserved [28]	KCCQ-CSS: 1.32 (0.45-2.19)
McMurray et al., 2019, DAPA-HF [29]	KCCQ-TSS: 1.18 (1.11-1.26), P < 0.001

The PRESERVED-HF [21] trial recruited HF patients with a mean EF of 60% and reported an improvement of 20.1 m over the placebo in a six-minute walk test (6MWT). However, dapagliflozin in the DEFINE-HF trial [22] did not show an increase in the distance walked in the 6MWT. The EMPERIAL-Preserved [23] trial did not find a functional improvement in patients with either reduced or preserved ejection fraction. The EMPA-TROPISM trial [24] investigated the effect of empagliflozin in nondiabetic patients with HFrEF for increasing exercise capacity, as shown by the cardiopulmonary exercise test and 6MWT with a significant improvement in the distance walked in six minutes. These results are shown in Table 6.

**Table 6:** Results of functional improvement of respective trials 6MWT: six-minute walk test; SGLT-2i: sodium-glucose transporter-2 inhibitor; NA: not available; m: meters; P: P* *value; EF: ejection fraction

Author, year, study	6MWT difference between SGLT-2i and placebo with 95% confidence interval (in meters)
Bhatt et al., 2021, SOLOIST-WHF [20]	NA
Nassif et al., 2021, DAPA-PRESERVED [21]	20.1 m (5.6-34.7), P = 0.007
Nassif et al., 2019, DEFINE-HF [22]	No statistically significant difference
Abraham et al., 2021, EMPERIAL [23]	-4.0 m (-16.0-6.0), P = 0.42; for EF <40%
Abraham et al., 2021, EMPERIAL [23]	4.0 m (-5.0-13.0), P = 0.37; for EF >40%
Santos-Gallego et al., 2021, EMPA-TROPISM [24]	116 m,* *P = <0.001
Jensen et al., 2019, EMPIRE-HF [25]	NA
Lee et al., 2021, SUGAR-DM-HF [26]	-9.9 m (-34.4-14.7), P = 0.43
Packer et al., 2020, EMPEROR-Reduced [27]	NA
Anker et al., 2021, EMPEROR-Preserved [28]	NA
McMurray et al., 2019, DAPA-HF [29]	NA

We employed the Review Manager software (RevMan 5.4.1, The Cochrane Collaboration, Copenhagen, Denmark) to perform the statistical analysis. The odds ratio (OR) and the 95% confidence interval (CI) for each analyzed parameter were calculated by using the fixed-effects model and the Mantel-Haenszel method. The OR of >1 favors increased risk of HHF/CVM and ACM, <1 indicates less risk of the same, and 1 indicates no observed association. The statistical significance was achieved with a P* *value of <0.05. The heterogeneity among the studies was calculated by using Higgins I^2^ with a value range of 0%-100% [33]. The I^2^ value of 0% reflects no heterogeneity, <25% mild, 25%-<50% moderate, 50%-<75% severe, and more than 75% very severe. We used the same software to draw forest plots for a visual impression of the results and funnel plots to reflect the publication bias in the studies.

**Figure 2:**
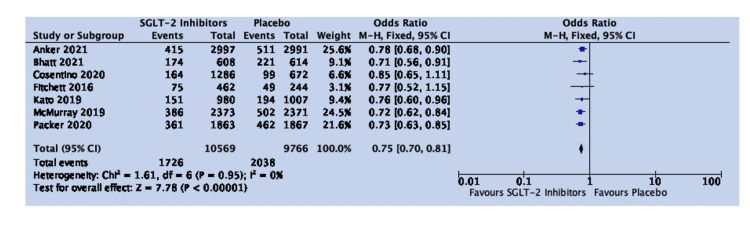
Forest plot for studies comparing SGLT-2 inhibitors and placebo for the composite of HHF/CVM in all cases of HF SGLT-2: sodium-glucose transporter-2; CI: confidence interval; HHF/CVM: hospitalization for heart failure or cardiovascular mortality; HF: heart failure; M-H: Mantel-Haenszel; df: degrees of freedom Sources: [19,20,27-29,31,32]

The results of 20,335 patients in seven studies showed a risk reduction in HHF/CVM in all patients with HF. The OR was 0.75, 95% CI was 0.70-0.81, P value was <0.00001, and heterogeneity (I^2^) was 0%. Sodium-glucose transporter-2 (SGLT-2) inhibitors are associated with a statistically significant risk reduction in HHF/CVM in HF patients (Figure 2).

**Figure 3:**
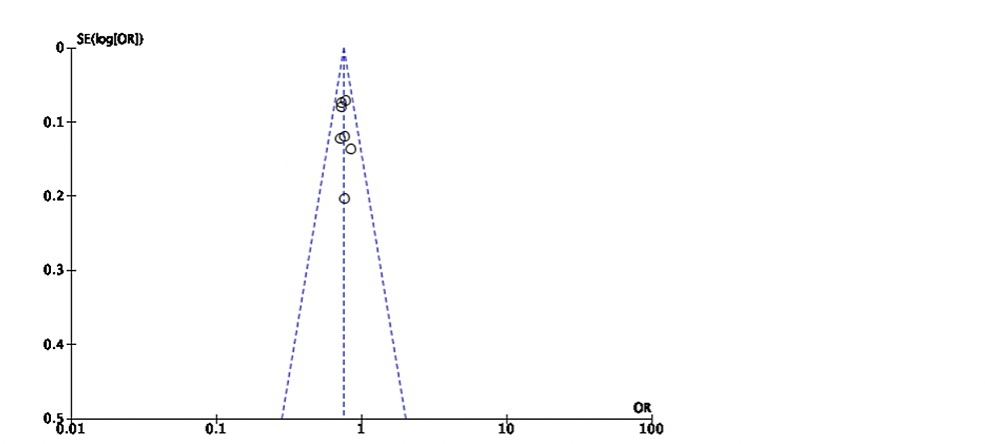
Funnel plot for studies comparing SGLT-2 inhibitors and placebo for the composite of HHF/CVM in all cases of HF SE: standard error; OR: odds ratio; SGLT-2: sodium-glucose transporter-2; HHF/CVM: hospitalization for heart failure or cardiovascular mortality; HF: heart failure Sources: [19,20,27-29,31,32]

No publication bias was seen in the seven studies involving 20,335 patients (Figure 3).

**Figure 4:**
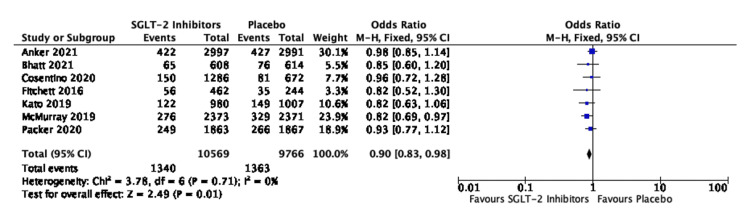
Forest plot for studies comparing SGLT-2 inhibitors and placebo for ACM in all cases of HF SGLT-2: sodium-glucose transporter-2; CI: confidence interval; ACM: all-cause mortality; HF: heart failure; M-H: Mantel-Haenszel; df: degrees of freedom Sources: [19,20,27-29,31,32]

There was a statistically significant reduction in the risk of ACM between the two groups with OR of 0.90, 95% CI of 0.83-0.98, and P value of 0.01. No heterogeneity bias was seen in the seven studies involving 20,335 patients (I^2^ = 0%) (Figure 4).

**Figure 5:**
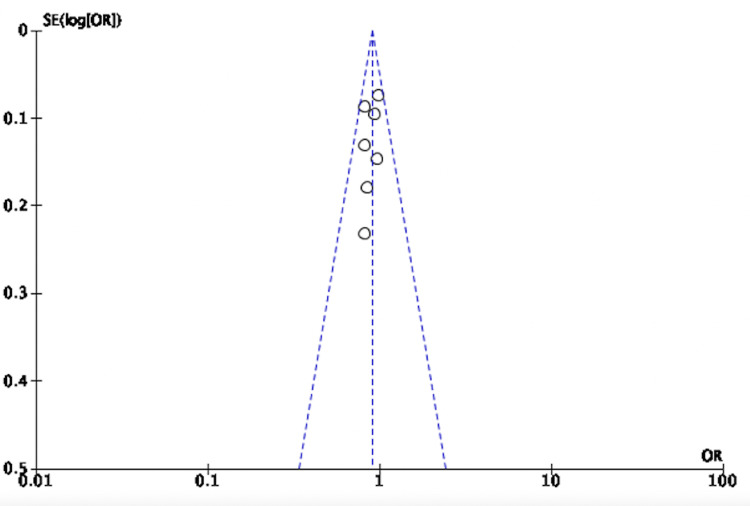
Funnel plot for studies comparing SGLT-2 inhibitors and placebo for ACM in all cases of HF SE: standard error; OR: odds ratio; SGLT-2: sodium-glucose transporter-2; ACM: all-cause mortality; HF: heart failure Sources: [19,20,27-29,31,32]

No publication bias was seen in the seven studies involving 20,335 patients (Figure 5).

**Figure 6:**
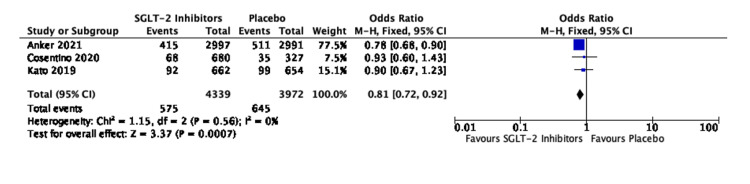
Forest plot for studies comparing SGLT-2 inhibitors and placebo for the composite of HHF/CVM in cases of HFpEF SGLT-2: sodium-glucose transporter-2; CI: confidence interval; HHF/CVM: hospitalization for heart failure or cardiovascular mortality; HFpEF: heart failure with preserved ejection fraction; M-H: Mantel-Haenszel; df: degrees of freedom Sources: [19,28,31]

The three studies evaluating 8,311 patients with HFpEF revealed an OR of 0.81, 95% CI of 0.72-0.92,* *and* *P value of 0.0007. These studies did not have heterogeneity (I^2^ = 0%) (Figure 6).

**Figure 7:**
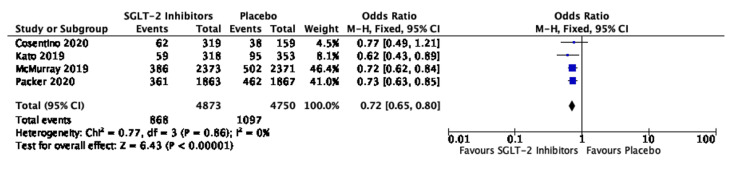
Forest plot for studies comparing SGLT-2 inhibitors and placebo for the composite of HHF/CVM in cases of HFrEF SGLT-2: sodium-glucose transporter-2; CI: confidence interval; HHF/CVM: hospitalization for heart failure or cardiovascular mortality; HFrEF: heart failure with reduced ejection fraction; M-H: Mantel-Haenszel; df: degrees of freedom Sources: [19,27,29,31]

The four studies evaluating 9,623 patients with HFrEF revealed an OR of 0.72, 95% CI of 0.65-0.80, and P value of 0.00001. No heterogeneity was seen in these studies (I^2^ = 0%) (Figure 7).

Discussion

Cardiovascular disease (CVD) is the most common cause of morbidity and mortality in any form of diabetes mellitus [34]. It may manifest as atherosclerotic coronary artery disease (ASCAD), peripheral vascular disease (PVD), cerebrovascular disease, or cardiomyopathy resulting to heart failure [35]. The presence of CVD almost doubles the inherent mortality rate of diabetes mellitus [36]. Coronary artery disease may lead to heart failure (HF), which can be the final evolutionary point of almost any cardiac pathology resulting in a compromised quality of life, more so after an episode of decompensation. One event of decompensated HF increases the risk of further hospitalizations and mortality [37].

These facts paved the way for conducting cardiovascular outcome trials (CVOTs) for new anti-diabetic drugs, which revealed the cardioprotective benefits of glucagon-like peptide 1 receptor agonists (GLP-1 RAs) and sodium-glucose transporter-2 (SGLT-2) inhibitors [38,39]. These studies demonstrated a decrease in the incidence of hospitalization for heart failure in patients treated with gliflozins. Further trials involving patients with HF, irrespective of their diabetic status, confirmed these findings [27,28,29]. Additionally, there is a need for assessment of symptomatic burden and quality of life (QoL) of HF patients despite promising results for event rates such as composite of hospitalization for heart failure or cardiovascular mortality (HHF/CVM) and irrespective of their diabetic status.

The symptom burden may be measured with the Kansas City Cardiomyopathy Questionnaire-12 (KCCQ-12). The KCCQ-12 is a 23-item, pathology-specific questionnaire answered by the patient before and after the study. It is a valid and reproducible tool for assessing the health status, including symptom burden, QoL, and physical and social functional status of HF patients. Various subgroups of KCCQ such as clinical summary score (KCCQ-CSS) quantify physical function and symptoms, total symptom score (KCCQ-TSS) includes the frequency and severity of symptoms, and overall summary score (KCCQ-OSS) measures total symptom score, QoL, social and physical function. The scores are transformed to a scale of 0-100, and higher scores signify better health [40]. The six-minute walk test (6MWT) investigates the distance covered by the patients in six minutes while walking at their maximum pace.

Some earlier studies involving beta-blockers, angiotensin-converting enzyme inhibitors (ACEi), angiotensin receptor neprilysin inhibitor (ARNI), or spironolactone failed to show any or revealed a minimal change in symptom burden or functional status [41,42]. One trial involving ivabradine and another instituting exercise therapy showed minor improvements in these scores [43,44]. The SGLT-2 inhibitors included in this meta-analysis decreased the decompensated HF risk and improved the symptomatic burden of the patient, as reflected in the increase in KCCQ scores.

The mechanism for this symptomatic improvement could be one multifold, a decrease in lung congestion as evidenced by a rapid lowering of pulmonary artery pressure and a decrease in preload [45]. It may also be due to a loss of the interstitial fluid rather than intravascular volume [46]. The increased ketogenesis allowing the myocardium to use ketones and improve its energetics is another factor [47]. This class of drugs reduces afterload and arterial stiffness, which could help reverse the remodeling of the failing heart [48,49]. Other possible events could be angiogenesis improving microcirculation and a more efficient myocytic mitochondrial function translating into increased cellular efficiency [50,51]. The weight loss resulting from SGLT-2i use would add to any of the above factors.

We observed a significant improvement in the symptom burden of HF patients in each of the studies [21-29] but only a modest increase in the distance walked during the 6MWT [21-23,24,26]. One possible explanation could be the presence of musculoskeletal (MSK) comorbidities [52]. Also, the 6MWT is an assessment done at one point in time and not a continuous one, which would be more reflective of the actual functional status of the patient. The EMPA-TROPISM trial investigated cardiopulmonary exercise testing, which evaluated for peak oxygen consumption (VO_2_), a parameter independent of the patient’s MSK restrictions. There was a statistically significant increase in its value [24].

A critical aspect of this study was the benefit enjoyed by the patients irrespective of their ejection fraction (EF). There were six trials studying patients with heart failure with preserved ejection fraction (HFpEF), four of which showed a decreased risk of HHF/CVM, and the other four revealed symptomatic improvement, with two having a significant increase in the distance walked by patients in the 6MWT. The treatment available for HFpEF so far has been diuretics to reduce pulmonary congestion with the unwanted activation of the renin-angiotensin-aldosterone system [53].

The incidence of adverse effects was low and not statistically significant. The CANVAS and DECLARE-TIMI 58 trials showed a statistically non-significant increased risk of amputation, fracture, and volume depletion but a statistically significant decrease in the incidence of acute kidney injury (AKI). The PRESERVED-HF, EMPA-REG Outcome, and DEFINE-HF trials reported an increased risk of volume depletion. The EMPEROR-Preserved trial had an increased risk of urinary tract infection (UTI) and fracture only, whereas the EMPEROR-Reduced trial showed a slight increase in the risk of fracture, amputation, UTI, and volume depletion. DAPA-HF patients were at increased risk for volume depletion but had less risk of AKI. There was an increased incidence of volume depletion in the EMPERIAL-Reduced trial and increased incidence of urogenital infections in the EMPERIAL-Preserved trial. An increased risk of diarrhea was seen in the SOLOIST-WHF trial. However, the appearance of any of these adverse events did not carry a statistical significance.

There are some limitations to our study. There was heterogeneity in the follow-up period of different trials. The sample size of trials in HFpEF patients was small except for EMPEROR-Preserved, which could affect the significance of the study. We included a few subgroup analyses whose randomization has not been validated, thus increasing the risk of bias. However, the baseline characteristics of HF subgroups in these trials revealed an apt balance between the two arms.

## Conclusions

This meta-analysis of more than 23,000 heart failure (HF) participants demonstrated an improvement in the symptom burden of HF patients when treated with sodium-glucose transporter-2 (SGLT-2) inhibitors. These observations add to the benefits seen in the risk of the composite of hospitalization for heart failure or cardiovascular mortality (HHF/CVM). The improvements in symptom burden, reduced risk of decompensated HF, and survival benefits span across all ranges of ejection fraction (EF) and patients’ diabetic status. These findings provide more evidence for the use of SGLT-2 inhibitors in managing broad categories of HF patients, especially in heart failure with preserved ejection fraction. It may help clinicians make an informed management choice for symptomatic and functional improvement of heart failure patients. We would need more studies reinforcing this review’s observations and focusing on the factors related to this improvement.
